# A second species of *Pseuduvaria* in China: the identity of the enigmatic species *Meiogyne kwangtungensis*

**DOI:** 10.3897/phytokeys.172.61025

**Published:** 2021-01-29

**Authors:** Qing-Long Wang, Hui Zhang, Yun-Yun Shao, Zhu-Nian Wang, Bine Xue

**Affiliations:** 1 Tropical Crops Genetic Resources Institute, CATAS, Haikou 571101, Hainan, China Tropical Crops Genetic Resources Institute Haikou China; 2 College of Horticulture and Landscape Architecture, Zhongkai University of Agriculture and Engineering, Guangzhou 510225, Guangdong, China Zhongkai University of Agriculture and Engineering Guangzhou China; 3 Guangdong Provincial Key Laboratory of Digital Botanical Garden, South China Botanical Garden, Chinese Academy of Sciences, Guangzhou 510650, Guangdong, China China Botanical Garden, Chinese Academy of Sciences Guangzhou China

**Keywords:** Annonaceae, *
Meiogyne
*, molecular phylogeny, morphology, *
Pseuduvaria
*

## Abstract

*Meiogyne kwangtungensis* is a rare species endemic to Hainan, China, known just from two fruiting collections made in the 1930s. Although it was published under the name *Meiogyne* in 1976, it was suggested that it might be better placed within *Pseuduvaria* or *Mitrephora*. For decades, this species was never collected again, thus its true generic affinity remained unresolved due to the lack of flowers. During a field exploration in Hainan, we re-discovered this species and collected a flowering specimen for the first time. The flower immediately confirmed its affinity with *Pseuduvaria*. Phylogenetic analyses of five chloroplast regions (*psbA-trnH*, *trnL-F*, *matK*, *rbcL*, and *atpB-rbcL*; ca. 4.2 kb, 70 accessions) also unambiguously placed *Meiogyne kwangtungensis* in the *Pseuduvaria* clade (PP = 1.00, ML BS = 99%). Morphologically, it is most similar to *P. multiovulata* which is endemic to Myanmar and Thailand, both with often-paired flowers, long pedicels and short peduncles, and often 1–2 monocarps. However, it differs in having smaller flowers with kidney-shaped glands on the inner petals, fewer stamens and carpels, smaller ovoid monocarps with an apicule and fewer seeds. On the basis of the combined molecular phylogenetic and morphological data, we propose a new combination, *Pseuduvaria kwangtungensis* (P.T.Li) Qing L.Wang & B.Xue. A full description including floral characters and a color plate are provided here for this species. A key to species in the genus *Pseuduvaria* in China is also provided.

## Introduction

The genus *Meiogyne* Miq. is a medium-sized genus in tribe Miliuseae Hook.f. & Thomson of Annonaceae (Chatrou et al. 2012; Thomas et al. 2012; Xue et al. 2014; Guo et al. 2017). It consists of ca. 30 species of trees or shrubs, distributed in wet tropical lowland and lower montane rainforests across South-east Asia and the western Pacific (Thomas et al. 2012; Tan et al. 2014; Xue et al. 2014, 2017; Turner and Utteridge 2015; Johnson et al. 2019). Species in *Meiogyne* are characterized by sub-equal petals, inner petals with corrugated or verrucose base of the adaxial surface and innermost stamens with tongue-shaped apical prolongations (van Heusden 1994; Thomas et al. 2012; Xue et al. 2014).

*Meiogyne kwangtungensis* Li was published in 1976, based on two fruiting collections (*F. C. How 73305*, IBSC, A, IBK and *Z. Huang 33693*, IBSC) from Hainan, China, in 1935 and 1933 respectively (Li 1976; Tsiang and Li 1979). After that, it was not collected again. The long fruiting pedicle of this species (up to 50 mm in length) is unusual in *Meiogyne*, as most *Meiogyne* species have short flowering and fruiting pedicels (usually less than 30 mm in length, except *Meiogyne chiangraiensis* Chalermglin & M.F.Liu; Johnson et al. 2019). Rainer and Chatrou (2006) indicate that this species can also belong to *Pseuduvaria* or *Mitrephora*. Flower characters are essential for generic delimitation in Annonaceae, and the three genera, *Meiogyne*, *Pseuduvaria* and *Mitrephora* can be easily distinguished on that basis (van Hesuden 1992; Su and Saunders 2006). Therefore, flowers are required to confirm the correct generic placement of this species (Li and Gilbert 2011).

For the past few years, we have carried out several field explorations in Hainan to search for this species. The explorations finally resulted in new collections of *Meiogyne kwangtungensis*, including flowers and fruits. Based on the mature flowers, we are able to confirm that *Meiogyne kwangtungensis* should be placed in *Pseuduvaria*.

*Pseuduvaria* is a genus widely distributed in continental SE Asia and Malesia, extending from Indochina and the Philippines to New Guinea and NE Australia (Su and Saunders 2006). The only comprehensive taxonomic monograph recognizes 52 species in the genus (Su and Saunders 2006). Three new species and one new combination in Su et al. (2010) and one new species in Turner (2010) bring the total species recognized in *Pseuduvaria* to 57. The flowers of *Pseuduvaria* are often unisexual and it’s unique in having inner petals apically connivent over the reproductive organs, forming a mitriform dome (Su and Saunders 2006; Su et al. 2008). Each inner petal is differentiated into a distinct blade and basal claw, which results in three lateral apertures between the petal claws, enabling access by floral visitors (Su and Saunders 2006). Moreover, the adaxial surface of the inner petals often bears one or two protruding glands (Su and Saunders 2006). In contrast, the flowers of *Meiogyne* are bisexual and the inner petals are spreading and corrugated at the base of the adaxial surface.

With the available flowering materials and silica-gel samples for DNA extraction, we clarify the generic placement of *Meiogyne kwangtungensis* based on morphological data and phylogenetic analysis in this study.

## Materials and methods

### Morphological studies

The morphological characters were examined based on the living plants and specimens kept in the HITBC, IBSC, IBK, and KUN herbaria. Comparisons were also made against published *Pseuduvaria* species in the monograph and recent papers (Su and Saunders 2006; Su et al. 2010; Turner 2010; Li and Gilbert 2011).

### Phylogenetic studies

Total DNA of the silica-gel dried material of *Meiogyne kwangtungensis* (*Q. L. Wang 20200528002*, IBSC) was extracted using a modified CTAB method (Doyle and Doyle 1987). Five chloroplast regions (*psbA-trnH*, *trnL-F*, *matK*, *rbcL*, and *atpB-rbcL*) were newly generated. For detailed information on PCR amplification and primer sequences we refer to Su et al. (2008). 54 *Pseuduvaria* species from Su et al. (2010) were included in this study. *Monocarpia euneura* Miq. and 14 species in the tribe Miliuseae were selected as outgroups based on the phylogenetic framework reported in previous studies (Chatrou et al. 2012; Chaowasku et al. 2014; Guo et al. 2017; Xue et al. 2018, 2020a). Sequences were downloaded from the nucleotide database of the National Centre for Biotechnology Information (http://www.ncbi.nlm.nih.gov). The final data matrix comprised a total of 70 species of Annonaceae. The information on sequence alignment can be found in Xue et al. (2018).

Detailed information about the samples, localities and GenBank accession numbers are all listed in the Appendix 1.

Phylogenetic analyses were done using Bayesian Inference (BI) and maximum likelihood (ML) methods. The information on model selection of the sequence matrix constructed could refer to Xue et al. (2018). The best partition scheme suggested five partitions based on DNA region identity with GTR + I + Γ chosen for *matK* and *rbcL*; and GTR + Γ selected for *atpB-rbcL*, *psbA-trnH* and *trnL-F* regions. Detailed methods for tree reconstruction could refer to Xue et al. (2018) and Xue et al. (2020b).

## Results

**Figure 1.**
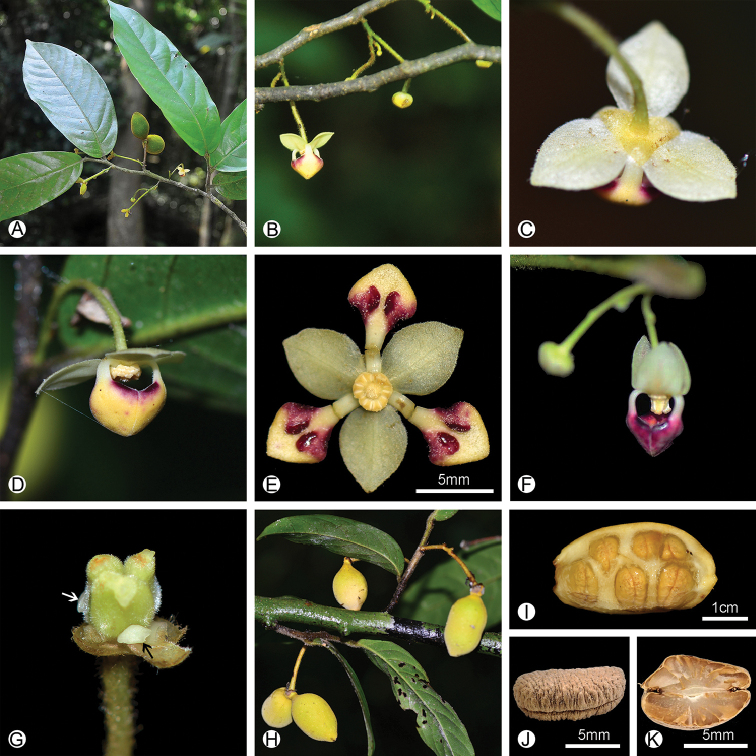
Morphology of *Pseuduvaria kwangtungensis*, comb. nov. **A** flowering branch **B** inflorescence **C** bottom view of a male flower **D** side view of a male flower **E** male flower, top view, inner petals manually separated to show adaxial inner petal surface with paired glands **F** a female flower **G** gynoecium of the female flower, showing three carpels and two staminodes (with black and white arrows) **H** fruits **I** inside of a monocarp, showing seeds in two series **J** Single dried seed, showing the grooved raphe **K** section of the seed, showing the spiniform endosperm rumination. Photos: Q. L. Wang (**A–I**); B. Xue (**J, K**).

**Figure 2.**
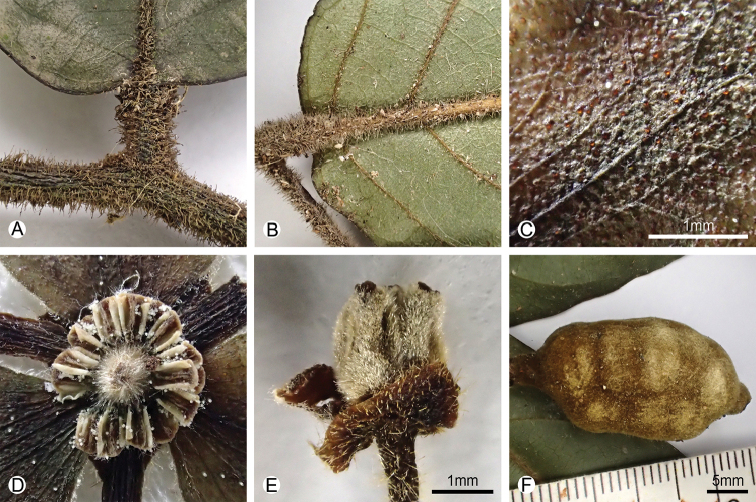
Specimen morphology of *Pseuduvaria kwangtungensis*, comb. nov. **A** adaxial view of the leaf base and the petiole **B** abaxial view of the leaf base and the petiole **C** close-up of the adaxial surface of the outer petal, showing the dense tiny golden glands **D** dried androecium, showing the morphology of the stamens **E** dried gynoecium, showing the hairy carpels **F** dried monocarp, showing the pubescent indumentum and the shallowly transversely constriction between seed. Photos: B. Xue.

The morphological observation is illustrated in Figs 1, 2, and discussed in detail below.

**Figure 3.**
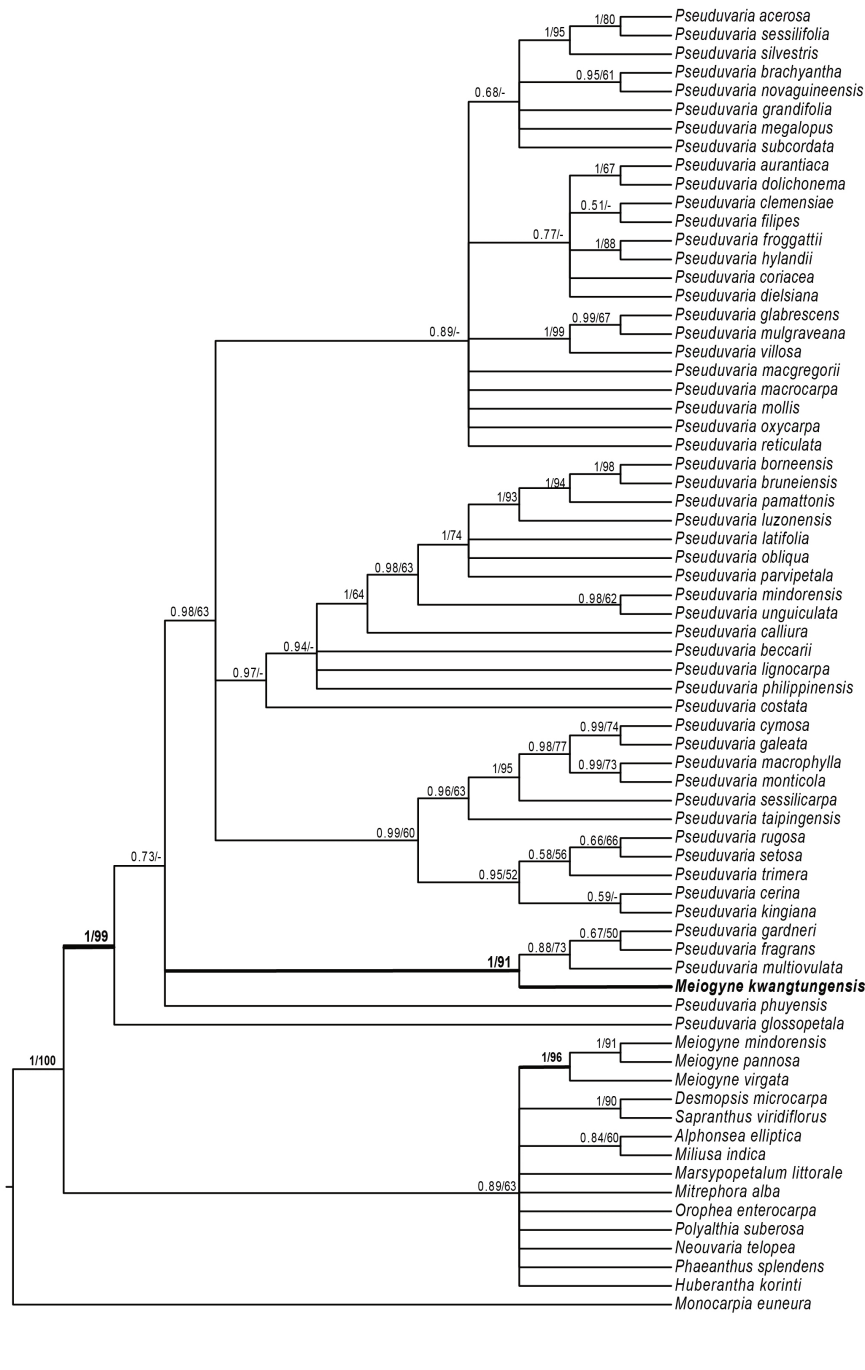
Bayesian 50% majority-rule consensus tree under partitioned models (cpDNA data: *atpB-rbcL*, *matK*, *psbA-trnH*, *rbcL*, and *trnL-F*; 70 taxa). Numbers at the nodes indicate Bayesian posterior probabilities and maximum likelihood bootstrap values (> 50%) in that order.

The concatenated alignment of the 70-taxon dataset consisted of 4,261 aligned positions (*psbA-trnH*: 430 bp, *trnL-F*: 891 bp, *matK*: 810 bp, *rbcL*: 1,343 bp, and *atpB-rbcL*: 787 bp). The Bayesian analyses and ML resulted in similar topologies. The 50% majority-rule consensus tree resulting from the Bayesian analyses under five-partitioned model is shown as Fig. 3.

The backbone of the tribe Miliuseae is not well resolved as in previous studies. The sampled *Pseuduvaria* species form a well-supported clade (PP = 1; ML BS = 99%; Fig. 3). The three *Meiogyne* species, viz. *Meiogyne mindorensis* (Merr.) Heusden, *M. pannosa* (Dalzell) J. Sinclair, and *M. virgata* (Blume) Miq. form a well-supported clade (PP = 1; ML BS = 96%; Fig. 3). *Meiogyne kwangtungensis*, however, is not retrieved in the same clade as the three *Meiogyne* species sampled, but nested within *Pseuduvaria* clade, and closely related to *Pseuduvaria gardneri* Y. C. F. Su, Chaowasku & R. M. K. Saunders, *P. fragrans* Y. C. F. Su, Chaowasku & R. M. K. Saunders and *P. multiovulata* (C. E. C. Fischer) J. Sinclair (PP =1; ML BS = 91%; Fig. 3).

## Discussion

With the new collections of the flowering specimens of *Meiogyne kwangtungensis*, the enigmatic identity of this species is resolved. The flowers of *Meiogyne kwangtungensis* are unisexual (Fig. 1D–G). Both female and male flowers are characterized by having longer inner petals that are apically connivent over the reproductive parts to form a mitriform dome (Fig. 1B, D, F). The inner petals are differentiated into distinct blades and claws, with the adaxial surface of the claw of the inner petal bearing two protruding glands (Fig. 1E). The stamen has a flat-topped connective extending over the thecae (‘uvarioid’ sesu Prantl 1891; Mols et al. 2004) (Fig. 1E, 2D). These characters are completely different from that of the *Meiogyne* species. In contrast, flowers of *Meiogyne* are bisexual; both whorls of petals are sub-equal and similar in shape; the inner petals are not connivent either. Therefore, the flower morphology of *Meiogyne kwangtungensis* is consistent with that of *Pseuduvaria*, which immediately confirmed its affinity with *Pseuduvaria*.

The molecular phylogeny further supported the placement of *Meiogyne kwangtungensis* in the genus *Pseuduvaria*. It falls into the same clade with *Pseuduvaria gardneri*, *P. fragrans* and *P. multiovulata* (PP =1.00; ML BS = 91%) (Fig. 3). Morphologically, *Meiogyne kwangtungensis* is most similar to *Pseuduvaria multiovulata* (C.E.C.Fischer) J.Sinclair in Thailand, both with 1–2 flowers per inflorescence, with long pedicels and short peduncles, and often with 1–2 monocarps (Su and Saunders 2006; Gardner et al. 2015). However, the two species differ in the size of the flowers, the shape of the inner petal glands, the number of stamens and carpels, the shape of the apex of the monocarps and the number of seeds per monocarp (Table 1). *Meiogyne kwangtungensis* has small flowers (outer petal ca. 7 mm long, inner petal ca. 8mm long) whereas *Pseuduvaria multiovulata* has larger flowers (outer petal 7.5–11 mm long, inner petal 9–18.5 mm long; Su and Saunders 2006). *Meiogyne kwangtungensis* has two kidney-shaped to ellipsoid glands on adaxial surface of the inner petals (Fig. 1E), whereas the inner petals glands of *Pseuduvaria multiovulata* are square (Su and Saunders 2006). *Meiogyne kwangtungensis* has 20–30 stamens in male flower and 3 carpels in female flower (Fig. 1E, G), whereas *Pseuduvaria multiovulata* has 110–115 stamens in male flower and ca. 11 carpels in female flower (Su and Saunders 2006). The monocarps of *Meiogyne kwangtungensis* have an apiculate apex, with 5–10 seeds per monocarp, whereas the monocarps of *Pseuduvaria multiovulata* do not have apicule, with ca. 17 seeds per monocarp (Su and Saunders 2006).

**Table 1.** Morphological comparison between *Pseuduvaria kwangtungensis* and *P. multiovulata*.

Characters	*P. kwangtungensis*	*P. multiovulata*
Length of the outer petals	ca. 7 mm long	7.5–11 mm long
Length of the inner petals	ca. 8 mm long	9–18.5 mm long
Shape of inner petal glands	kidney-shaped to ellipsoid	square
Number of stamens	20–30	110–115
Number of carpels	3	ca. 11
Shape of the apex of the monocarps	apiculate	do not have apicule
Number of seeds per monocarp	5–10	ca. 17

**Figure 4.**
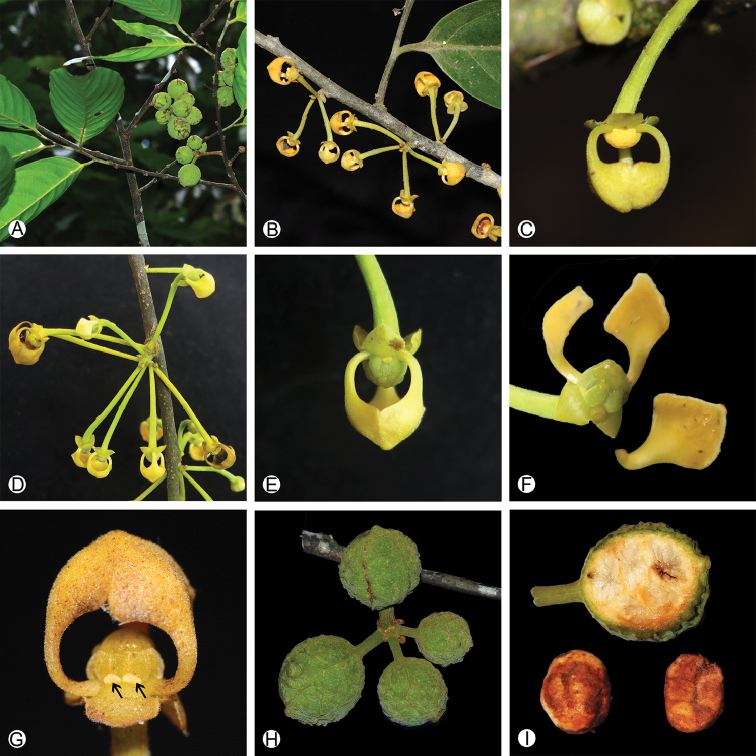
Morphology of *Pseuduvaria trimera***A** fruiting branch **B** male inflorescence **C** side view of a male flower **D** female inflorescence **E** side view of a female flower **F** a female flower, showing the morphology of the inner petals and no glands on the inner petal **G** side view of the female flower, showing several carpels and two staminodes (with arrows) **H** fruits **I** section of one monocarp and seed morphology. Photos: Daniel Thomas (**A, H, I**); Yun-yun Shao (**B–G**).

In China, only one *Pseuduvaria* species is recorded in Yunnan Province, i.e. *Pseuduvaria trimera* (Craib) Y.C.F.Su & R.M.K.Saunders (Li and Gilbert 2011) (Fig. 4). This species is relatively widely distributed, also occurring in Myanmar, Thailand and Vietnam. *Meiogyne kwangtungensis* and *Pseuduvaria trimera* could be easily differentiated from each other by the growth habit, the morphology of leaf, inflorescence, flower and fruit. *Pseuduvaria trimera* is a tree up to 20 m tall (Su and Saunders 2006; Li and Gilbert 2011), whereas *Meiogyne kwangtungensis* is a shrub to 4 m tall. The leaf laminas of *Pseuduvaria trimera* are subcoiaceous with 14–18 pairs of secondary veins (Fig. 4A), whereas leaf laminas of *Meiogyne kwangtungensis* are papery with ca. 10 pairs of secondary veins (Fig. 1A). The inflorescences of *Pseuduvaria trimera* are clustered on young branches with yellow flowers (Fig. 4B–G), whereas those of *Meiogyne kwangtungensis* are axillary with cream-colored or purple flowers (Fig. 1B–F). *Pseuduvaria trimera* is distinct in lacking any glands on the clawed inner petals (Fig. 4F), whereas *Meiogyne kwangtungensis* has a pair of glands on the adaxial surface of the inner petals (Fig. 1E). *Pseuduvaria trimera* has globose, stipitate monocarps with rugulose pericarps (Fig. 4H, I; Su and Saunders 2006; Li and Gilbert 2011), whereas *Meiogyne kwangtungensis* has ovoid, sessile monocarps with smooth pericarp (Fig. 1H).

*Meiogyne kwangtungensis* is different from all species in *Pseuduvaria*. Both the morphological and molecular phylogenetic data support the transfer of *Meiogyne kwangtungensis* to *Pseuduvaria*, thus a new nomenclatural combination is proposed here. Additionally, as the name *Meiogyne kwangtungensis* was published based on only two fruiting collections lacking flowers, we provide a detailed description of the flower morphology and an updated description for the fruit morphology. A key to the two *Pseuduvaria* species in China is also provided.

### Taxonomic treatment

#### Pseuduvaria kwangtungensis

Taxon classificationPlantaeMagnolialesAnnonaceae

(P.T.Li) Qing L.Wang & B.Xue comb. nov.

4FA7CCE5-DDFC-58B9-857D-84DFAF7252C1

urn:lsid:ipni.org:names:77214765-1

Figs 1 , 2

##### Chinese name.

hai nan jin gou hua (海南金钩花)

##### Basionym.

*Meiogyne kwangtungensis* P.T.Li, Acta Phytotax. Sin. 14(1): 104. 1976.

##### Type.

China. Hainan: Bao-ting Hisen, Xing-long, 25 Jul. 1935, *F. C. How 73305* (holotype: IBSC! [IBSC0003357]; isotypes, A [A00066602, photo!], IBK![IBK00190122], SN!).

##### Description.

***Shrubs*** to 3–4 m tall, d.b.h. ca. 5 cm. Monoecious. ***Branches*** black, densely villous when young, glabrescent (Fig. 1A). Petiole 2–3 mm, villous (Figs 1A, 2A); ***leaf blade*** oblong to elliptic, 6–18 × 2.5–5.5 cm, papery, adaxially glossy and glabrous except for pubescent midrib (Figs 1A, 2A), abaxially glaucous and villous (Figs 1A, 2B), midvein adaxially impressed, secondary veins ca. 10 on each side of midvein and prominent on both surfaces, base rounded to sometimes shallowly cordate, apex acuminate (Fig. 1A). ***Inflorescences*** axillary, with up to 2–3 flowers, only one flower at anthesis per inflorescence (Fig. 1A, B). Flowering peduncles 3–10 mm long, ca. 1 mm in diameter, villous (Fig. 1A, B). Sympodial rachides inconspicuous (often less than 5 mm), internodes poorly developed with several bracts. Flowering pedicels 15–30 mm long, ca. 1 mm in diameter, densely villous with erect hairs (Fig. 1B, C). ***Sepals*** partially connate, triangular to ovate, ca. 2 mm long, ca. 2 mm wide, glabrous adaxially, densely puberulous with appressed hairs abaxially (Fig. 1C). ***Outer petals*** ca. 7 mm long, ca. 5 mm wide, thin, broadly circular, glabrous adaxially, puberulous with appressed hairs abaxially and on the edge, cream-colored, without claws, dried with dense tiny golden glands adaxially (Figs 1C–E, 2C). ***Inner petals*** ca. 8 mm long, 4 mm wide, rhombic, apex acute, base acute, ca. 1 mm thick, very densely puberulous with appressed hairs adaxially, densely puberulous with appressed hairs abaxially, cream-colored with purple tinge on adaxial surface of the blade in staminate flower (Fig. 1D, E), and purple in pistillate flower (Fig. 1F); basal claw ca. 3–4 mm long; glands paired on adaxial surface of inner petal, kidney-shaped to ellipsoid, surface smooth, raised (Fig. 1E); apical aperture absent. Flowers unisexual. ***Staminate flowers*** with androecium ca. 1 mm long, ca. 2 mm wide; stamens ca. 20–30 per flower, ca. 0.9 mm long, ca. 0.7 mm wide (Fig. 2D). ***Pistillate flowers*** with gynoecium ca. 1.5 mm long, ca. 1.3 mm wide; carpels 3 per flower, ca. 1.2 mm long, ca. 0.5 mm wide (Figs 1F, G, 2E); ovules ca. 6–10 per carpel, bi-seriate; staminodes two (Fig. 1G). ***Fruiting*** peduncle 5–10 mm long, fruiting pedicel 20–50 mm (Fig. 1H). ***Monocarps*** 1–3, sessile or stipes to 3 mm long, ovoid, 20–37 cm long, 20–25 cm wide, very shallowly transversely constricted between seed when dry, densely tomentose, base rounded, apex apiculate (Figs 1H, 2F). ***Seeds*** 5–10 per monocarp, in 2 series, yellowish, semi-lenticular to ellipsoid, 12 to 14 mm long, 5–8 mm wide, 3–5 mm high, with rugose and pitted testa and a grooved raphe that is more or less straight (Fig. 1I, K), endosperm rumination spiniform (Fig. 1L).

##### Distribution and habitat.

Known from several localities in Hainan province: Bai-cha Mountain in San-ya and Xing-long in Bao-ting, growing in rain forests, open woodland in valleys, at low elevations (ca. 600 m a.s.l).

##### Phenology.

Flowering from March to June; fruiting from June to August.

##### Additional specimens examined.

China. Hainan: San-ya, Bai-cha Mountain, 13 Aug. 1933, *Z. Huang 33693* (IBSC0078951, SN); Bao-ting Hisen, Qi-xian Mountain, on mountain slopes under forest, alt. 549 m, 25 Apr. 2020, *Q. L. Wang BT20200425001* (ATCH, IBSC); alt. 584 m, 28 May 2020, *Q. L. Wang BT20200528001*, *BT20200528002* (ATCH, IBSC).

##### Preliminary IUCN conservation status.

CR D (IUCN 2012). *Pseuduvaria kwangtungensis* was assessed as CR D by Qin et al. (2017). Prior to this study, *P. kwangtungensis* was only represented in herbaria by two collections from Hainan, China, collected in 1933 and 1935 respectively. One of the authors, Dr. Qing-Long Wang, has undertaken extensive field surveys in Hainan, and only found this species again in two localities in Qi-xian Mountain in Bao-ting, with three and four mature individuals respectively. Although it’s possible more individuals may be discovered with more extensive field surveys, we intended to maintain the CR category.

### Key to *Pseuduvaria* in China

**Table:** 

1	Shrub to 4 m tall, d.b.h. ca. 5 cm. Leaf laminas membranous, secondary veins ca. 10 pairs. Inflorescences axillary, with up to 2–3 flowers; flowers cream-colored or purple, glands paired on adaxial surface of inner petal; carpels 3 per flower, stamens 20–30 per flower. Fruits with 1–3 monocarps. Monocarps ovoid, apex apiculate, smooth; sessile or stipes to 3 mm long. Distributed in Hainan	***P. kwangtungensis***
–	Trees to ca. 20 m tall, d.b.h. ca. 42 cm. Leaf laminas subcoiaceous, secondary veins 14–18 pairs. Inflorescences clustered (3–6) on young branches, each with 1–2 flowers; flowers yellow or light green; inner petals lack gland; carpels 7–14 per flower, stamens 45–56 per flower. Fruits with 7–8 monocarps. Monocarps globose, apex slightly apiculate, rugulose; stipes 10–14 mm long. Distributed in Yunnan	***P. trimera***

## Supplementary Material

XML Treatment for Pseuduvaria kwangtungensis

## Appendix 1

Voucher information and GenBank accession numbers for samples used in this study (—, missing data; *, newly generated sequences). Voucher data are given for accessions for which DNA sequences were newly obtained, using the following format: species, origin, voucher and Genbank accession numbers for *atpB-rbcL*, *matK*, *psbA-trnH*, *rbcL*, and *trnL-F*. For DNA sequences published in previous studies, voucher information is available from GenBank.

*Alphonsea elliptica* Hook. f. & Thomson, —, AY518807, JQ690402, —, AY319078; *Desmopsis microcarpa* R.E.Fr., —, AY518804, AY84146, AY319059, AY319173; *Huberantha korinti* (Dunal) Chaowasku, EU522345, EU522234, EU522124, EU522289, EU522179; *Marsypopetalum littorale* (Blume) B.Xue & R.M.K.Saunders, —, AY518835, JX544804, —, AY319140; *Meiogyne mindorensis* (Merr.) Heusden, —, JQ723776, —, JQ723863, JQ723916; *Meiogyne pannosa* (Dalzell) J.Sinclair, —, JQ723778, —, JQ723865, JQ723918; *Meiogyne virgata* (Blume) Miq., —, AY518798, JX544784, —, AY319094; *Miliusa indica* Lesch.ex A.DC., —, JQ723781, —, JQ723868, JQ723921; *Mitrephora alba* Ridl., —, AY518855, JQ889978, —, AY319106; *Monocarpia euneura* Miq., AY841381, AY518865, AY841477, —, AY319111; *Neo-uvaria telopea* Chaowasku, —, JX544751, JX544791, JX544755, JX544783; *Orophea enterocarpa* Maingay ex Hook.f. & Thomson, —, AY518815, JQ690417, —, AY319119; *Phaeanthus splendens* Miq., —, AY518864, JX544790, JX544754, AY319126; *Polyalthia suberosa* (Roxb.) Thwaites, AY841386, AY220439, AY841502, AY238956, AY319152; *Pseuduvaria acerosa* Y.C.F.Su & R.M.K.Saunders, EU522347, EU522236, EU522126, EU522291, EU522181; *Pseuduvaria aurantiaca* (Miq.) Merr., EU522348, EU522237, EU522127, EU522292, EU522182; *Pseuduvaria beccarii* (Scheff.) J.Sinclair, EU522349, EU522238, EU522128, EU522293, EU522183; *Pseuduvaria borneensis* Y.C.F.Su & R.M.K.Saunders, EU522350, EU522239, EU522129, EU522294, EU522184; *Pseuduvaria brachyantha* Y.C.F.Su & R.M.K.Saunders, EU522351, AY518837, EU522130, EU522295, AY319160; *Pseuduvaria bruneiensis* Y.C.F.Su & R.M.K.Saunders, EU522352, EU522241, EU522131, EU522296, EU522186; *Pseuduvaria calliura* Airy Shaw, EU522353, EU522242, EU522132, EU522297, EU522187; *Pseuduvaria cerina* J.Sinclair, EU522354, EU522243, EU522133, EU522298, EU522188; *Pseuduvaria clemensiae* Y.C.F.Su & R.M.K.Saunders, EU522355, EU522244, EU522134, EU522299, EU522189; *Pseuduvaria coriacea* Y.C.F.Su & R.M.K.Saunders, EU522356, AY518838, EU522135, EU522300, AY319161; *Pseuduvaria costata* (Scheff.) J.Sinclair, EU522357, EU522246, EU522136, EU522301, EU522191; *Pseuduvaria cymosa* (J.Sinclair) Y.C.F.Su & R.M.K.Saunders, EU522358, EU522247, EU522137, EU522302, EU522192; *Pseuduvaria dielsiana* (Lauterb.) J.Sinclair, EU522359, EU522248, EU522138, EU522303, EU522193; *Pseuduvaria dolichonema* (Diels) J.Sinclair, EU522360, EU522249, EU522139, EU522304, EU522194; *Pseuduvaria filipes* (Lauterb. & K.Schum.) J.Sinclair, EU522361, EU522250, EU522140, EU522305, EU522195; *Pseuduvaria fragrans* Y.C.F.Su, Chaowasku & R.M.K.Saunders, EU522397, EU522286, EU522176, EU522341, EU522231; *Pseuduvaria froggattii* (F.Muell.) Jessup, EU522362, EU522251, EU522141, EU522306, EU522196; *Pseuduvaria galeata* J.Sinclair, EU522363, EU522252, EU522142, EU522307, EU522197; *Pseuduvaria gardneri* Y.C.F.Su, Chaowasku & R.M.K.Saunders, GQ174302, GQ174298, GQ174294, GQ174300, GQ174296; *Pseuduvaria glabrescens* (Jessup) Y.C.F.Su & R.M.K.Saunders, EU522364, EU522253, EU522143, EU522308, EU522198; *Pseuduvaria glossopetala* Y.C.F.Su & R.M.K.Saunders, GQ174303, GQ174299, GQ174295, GQ174301, GQ174297; *Pseuduvaria grandifolia* (Warb.) J.Sinclair, EU522365, EU522254, EU522144, EU522309, EU522199; *Pseuduvaria hylandii* Jessup, EU522366, EU522255, EU522145, EU522310, EU522200; *Pseuduvaria kingiana* Y.C.F.Su & R.M.K.Saunders, EU522367, EU522256, EU522146, EU522311, EU522201; *Pseuduvaria kwangtungensis* (P.T.Li) Qing L.Wang & B.Xue [= *Meiogyne kwangtungensis* Li], China, Hainan, *Q. L. Wang 20200528002* (IBSC), MW415929*, MW415930*, MW415931*, MW415932*, MW415933*; *Pseuduvaria latifolia* (Blume) Bakh.f., EU522368, EU522257, EU522147, EU522312, EU522202; *Pseuduvaria lignocarpa* J.Sinclair, EU522369, EU522258, EU522148, EU522313, EU522203; *Pseuduvaria luzonensis* (Merr.) Y.C.F.Su & R.M.K.Saunders, EU522370, EU522259, EU522149, EU522314, EU522204; *Pseuduvaria macgregorii* Merr., EU522371, EU522260, EU522150, EU522315, EU522205; *Pseuduvaria macrocarpa* (Burck) Y.C.F.Su & R.M.K.Saunders, EU522372, EU522261, EU522151, EU522316, EU522206; *Pseuduvaria macrophylla* (Oliv.) Merr, EU522373, EU522262, EU522152, EU522317, EU522207; *Pseuduvaria megalopus* (K.Schum.) Y.C.F.Su & Mols 16235, EU522374, EU522263, EU522153, EU522318, EU522208; *Pseuduvaria mindorensis* Y.C.F.Su & R.M.K.Saunders, EU522375, EU522264, EU522154, EU522319, EU522209; *Pseuduvaria mollis* (Warb.) J.Sinclair, EU522376, EU522265, EU522155, EU522320, EU522210; *Pseuduvaria monticola* J.Sinclair, EU522377, EU522266, EU522156, EU522321, EU522211; *Pseuduvaria mulgraveana* Jessup, EU522378, EU522267, EU522157, EU522322, EU522212; *Pseuduvaria multiovulata* (C.E.C.Fisch.) J.Sinclair, EU522379, EU522268, EU522158, EU522323, EU522213; *Pseuduvaria nova—guineensis* J.Sinclair, EU522380, EU522269, EU522159, EU522324, EU522214; *Pseuduvaria obliqua* Y.C.F.Su & R.M.K.Saunders, EU522381, EU522270, EU522160, EU522325, EU522215; *Pseuduvaria oxycarpa* (Boerl.ex Koord.) Y.C.F.Su & R.M.K.Saunders, EU522382, EU522271, EU522161, EU522326, EU522216; *Pseuduvaria pamattonis* (Miq.) Y.C.F.Su & R.M.K.Saunders, EU522383, AY518840, EU522162, EU522327, AY319163; *Pseuduvaria parvipetala* Y.C.F.Su & R.M.K.Saunders, EU522384, EU522273, EU522163, EU522328, EU522218; *Pseuduvaria philippinensis* Merr., EU522385, EU522274, EU522164, EU522329, EU522219; *Pseuduvaria phuyensis* (R.M.K.Saunders, Y.C.F.Su & Chalermglin) Y. C. F. Su & R. M. K. Saunders, EU522342, AY518841, EU522121, EU522287, AY319114; *Pseuduvaria reticulata* (Blume) Miq., EU522386, EU522275, EU522165, EU522330, EU522220; *Pseuduvaria rugosa* (Blume) Merr., EU522387, AY518839, EU522166, EU522331, AY319162; *Pseuduvaria sessilicarpa* (J.Sinclair) Y.C.F.Su & R.M.K.Saunders, EU522388, EU522277, EU522167, EU522332, EU522222; *Pseuduvaria sessilifolia* J.Sinclair, EU522389, EU522278, EU522168, EU522333, EU522223; *Pseuduvaria setosa* (King) J.Sinclair, EU522390, EU522279, EU522169, EU522334, EU522224; *Pseuduvaria silvestris* (Diels) J.Sinclair, EU522391, EU522280, EU522170, EU522335, EU522225; *Pseuduvaria subcordata* Y.C.F.Su & R.M.K.Saunders, EU522392, EU522281, EU522171, EU522336, EU522226; *Pseuduvaria taipingensis* J.Sinclair, EU522393, EU522282, EU522172, EU522337, EU522227; *Pseuduvaria trimera* (Craib) Y.C.F.Su & R.M.K.Saunders, EU522394, EU522283, EU522173, EU522338, EU522228; *Pseuduvaria unguiculata* (Elmer) Y.C.F.Su & R.M.K.Saunders, EU522395, EU522284, EU522174, EU522339, EU522229; *Pseuduvaria villosa* Jessup, EU522396, EU522285, EU522175, EU522340, EU522230; *Sapranthus viridiflorus* G.E.Schatz, AY841391, AY743493, AY841515, JQ590194, AY319165.
